# Fighting spirit expressed by women with breast cancer in professional-and peer-led online support groups: effects on outcomes

**DOI:** 10.18689/mjcsr-1000102

**Published:** 2017-10-30

**Authors:** Morton A Lieberman, Stephen Lepore

**Affiliations:** 1 University of California San Francisco, USA; 2 Temple University, Philadelphia, PA, USA

**Keywords:** Comparison professional and peer led groups, online, fight attitudes, measured directly from group member interaction, outcomes depression

## Abstract

**Objective:**

Does fighting spirit reduce depression in women with breast cancer (BC)?

**Methods:**

Unlike previous studies using questionnaires, we assessed fighting spirit by analyzing interaction in BC support groups. Three online groups were studied: synchronous professionally led groups (Life Beyond Cancer), asynchronous professionallyled groups (Bosom Buddies) and asynchronous peer-led bulletin board groups (Peer Bulletin Boards). The samples were predominantly Caucasian, married, highly educated, and had early stage cancer. We used a content analysis program, DTSearch, to measure fighting words expressed in the groups. Linear regression was used to examine the relation between fighting words and follow-up depression, controlling for baseline depression and total word count. We also compared samples on the number of fight words expressed.

**Results:**

We found that viewing their cancer as a fight or battle would result in lowered depression was disconfirmed in all samples. The peer groups had twice as many fight words compared to professionally led groups.

**Conclusion:**

Previous studies as well as the current one strongly support the null hypothesis. Our finding based on a behavioral measure of fighting spirit does add to previous studies results. Unfortunately the common lay perspective for cancer sufferers is that not having fighting spirit would mean a psychological incapacity. The message is that “brave and good people defeat cancer and that cowardly and undeserving people allow it to kill them”. This view can create guilt, self flagellation and stress in woman already facing a difficult disease.

## Introduction

Much of the past research on the efficacy of fighting spirit to enhance survival and well being used a self report questionnaire to assess the independent variable. The present study was undertaken to examine once again fighting spirit using a unique measure, an analysis of breast cancer (BC) women’s comments in interaction with others in breast cancer support groups. Fighting spirit was coded from their group’s protocols.

Possessing a fighting spirit for women with breast cancer has become an all too common mantra. The belief is that the progression and outcomes of cancer can be influenced by this attitude. Fighting spirit is characterized by optimistically viewing cancer as a challenge and having a determination to fight the cancer and not allow it to disrupt lives. This view is embedded in cultural beliefs and promoted by the popular media that see cancer as a life-threatening disease that can be defeated by a woman’s character [1]. Fund-raising marches for breast cancer, numerous articles in women’s magazines and some of the private funders of breast cancer research all reflect this attitude despite little scientific basis for these beliefs.

### Background

An early study [2] of 57 patients found that those having a high fighting spirit were more likely to be disease-free and survive for 5 to 10 years. A larger follow-up study (N = 578) that used the self-report measure first developed in the aforementioned study failed to find a positive relationship between fighting spirit and survival [3]. A review [4] of the scholarly literature on positive psychology and cancer found 12 studies examining the prognostic value of fighting spirit for cancer survival. Most of these 12 showed negative findings. The authors of this review concluded: people with cancer should not feel pressured into adopting particular coping styles to improve survival or reduce the risk of a recurrence. A subsequent study by Watson et al. [5], not included in their review, found again that fighting spirit offered no advantage for survival.

## The Study

### Sample

We opted for a wide range of BC online groups; 1) synchronous professionally led groups, Life beyond Cancer [6]; the other two were asynchronous; 2) Bosom Buddy [7] study that used the bulletin board format with a professional leader and 3) peer led bulletin boards [8]. All three of these studies yielded significant reductions in depression post group.

#### Bosom Buddies

Seventy-two women with primary breast carcinoma were assigned randomly to a 12-week, web-based, social support group with a waitlist (usual care) control group. The group was semi-structured, moderated by a health care professional, and delivered asynchronous. The program introduced a new topic related to breast cancer each week. Eighty percent of the participants identified themselves as Caucasian. The average age of participants was 49.5 years old. Sixty-eight percent were married, Most of the participants were highly educated: 36% had attended at least some graduate school, 28% had completed a bachelor’s degree, 28% had attended some college. Intervention was associated with reduced depression and cancer-related trauma, as well as lower perceived stress, with effect sizes that ranged from 38 to 0.54.

#### Life Beyond Cancer:

A two-armed randomized controlled trial with one-month pretest and posttest assessments was conducted. Non-metastatic breast cancer survivors (N = 184) with elevated distress were randomized to either a facilitated experimental “prosocial” internet support group (P-ISG) or a facilitated experimental “prosocial” internet support group (P-ISG) comparison condition. The P-ISG intervention included allelementsofthestandard (S-ISG), including six professionally- facilitated live chat sessions (90-minute weekly sessions) and access to an asynchronous discussion board, but with critical additions directed toward increasing helping others. Both in the experimental and control condition, the professional leaders followed a manual. Ninety-five percent of the participants identified themselves as Caucasian. The average age of participants was 52.7 years old. Eighty five percent were married. Most of the participants were highly educated: 51% high school graduate 49% college graduate. Relative to the P-ISG, participants in the S-ISG intervention had a lower level of depression and anxiety symptoms post intervention.

#### Peer Bulletin Boards:

Upon joining a group and 6 months later, new members (N = 114) to breast cancer bulletin boards (N=5) completed self-report measures of depression scale, posttraumatic growth, psychosocial well-being, and quality of life. The study required participants to fill out four on-line questionnaires upon joining the study and again six months later. One hundred and fourteen women were recruited from the five boards. They had a mean age of 46.2 years. Participants joined the board on average 10 months from their date of diagnosis, with a median of 3 months. Forty six percent had attained a college or graduate degree, while 9 percent had attained only a high school diploma. Finally, 38 percent of participants identified as stage I, 37 percent and 21 percent identified as stage II and III respectively and 4 percent identified as stage IV, providing a distribution of breast cancer diagnoses. We compared time 1 scores between those who completed the 6-month follow-up with those who did not. There were no differences between the groups in measures of outcomes and demographic variables. Three mediators were used as covariates in changes in the outcome measures: stage, clinical depression at time 1 and the number of months they posted over the 6-month pre-post data collection. For analytic purposes, participation was divided into three categories: 1–2 months, 3–4 and 5–6. To test for the effects of depression at the onset of the study on our measures of change, we divided the sample into not clinically depressed and clinically depressed (CES-D [9] score > 16). To test for the effects of severity of cancer, the sample was divided into cancer stages. To test for change over the six months, repeated measures MANOVA was run with the three outcome measures and the three mediators as covariates to test for interaction. Overall, participants improved in all three measures of quality of life after six months. Significant interaction was found for all three mediators.

## Procedure

As indicated, this study utilized three samples drawn from our previous studies. Included was a peer led breast cancer bulletin board, a professionally led chat room, a professionally facilitated bulletin board. For the study reported here outcomes in all the studies used measures of depression, the CES-D [9] in two of the studies and the HADS [10] in one. In all of the studies, time one measures were assessed prior to participants entering the intervention group and time two was assessed after the intervention, with the exception of the newsgroups which use an arbitrary six months since the participant joined the bulletin board because they joined at different times.

The independent variable was the number of times each participant’s posting contained fighting words. We used a content analysis program, dtSearch (dtSearch.com). In the analysis we controlled for the number of words each woman posted in the group.

The analytic strategy was using, for each of the samples, a linear regression: regressing follow-up depression scores on: 1)*T1* CES-D or HADS, 2) total word count and 3) the number of fight words (Table2). Prior to these regressions we compared the samples on the number of fight words (Table1) and used an analysis of variance and contrasts, with the two professionally led groups compared to the peer groups.

## Example of Use of Fighting Words

These were drawn from all three samples:

Could be so much worse than this little discomfort. I only have to read about some of the problems here to see that. I am awed by the courage and **fighting** spirit of the women here! It’s always hard to lose a friend, even more so to lose one to the same monster we’re **fighting**. Take comfort that you were there for her when she needed you, and I’ll bet she knows tonight that she has not been forgotten.

Oh, L, isn’t it enough that we’ve had to **battle** this, without our sisters having to go through it, too? See above to S., same goes to you! Every person does their **battle** with this beast in their own way, but we ALL need the love and support of our families and friends, even when we don’t know we do. SMILE So AC, I’m grateful that I kept some. As to being grounded, thanks, I don’t always feel that way. I feel ugly and funny looking, but my body is **fighting** back! In a year, I’ll look normal again, until then, I ignore the way I look… well…mostly. You are a strong person. I would go stir crazy, staying. Sigh Probably not even really the insurance company’s fault…but I really needed a place to scream about it. Good for you DÜ! We need to continue to **fight** to understand all billing, and who should be paying it. And make sure that it is paid by the right people. To read of all you wonderful women **fighting** and under-going today’s treatments makes me want to reach out and hug you all and let you know that it is only the special people, with a strange but this makes sense to me! I had my mastectomy in ‘92 then a reoccurrence on the same side in ‘96. Treatment has changed, but I’m still here and **fighting**. I have recently had a hysterectomy as it had migrated downwards, though was contained. I don’t know if I will be having anymore treatment now as when I went remember to change it if I decide to go for this! Welcome D. The waiting is the hardest part. Once things begin to happen it is easier to feel the **fight** has begun and you are being active. As we are a group from around the world, there is usually someone around here! This is the really good side of the wisdom and love. There are few places on the net as safe. This must make it very tough for you, but you are an individual and so is your cancer. **Battle** it, rant and rave here and you’ll feel better and how you feel mentally is very important.

My cancer is genetic. My mastectomy was in March! I’m It is hard, but you have to put yourself out there and tell people up front that this is what you have and that you are fighting, but you treasure them and would appreciate their support and help - you are still you, the same as always, just not going to be quite as active as usual for you.

P, so I am so pleased that some angels have stepped in to help you with your travel! It will seem unreal P until you feel the **‘fight’** has begun, but now you have the date you can prepare yourself a little, getting in plenty of supplies of chocolate, sorting out books or sewing etc. Doing bits is why we have regular check-ups! What a bummer A. but I am glad to see you are moving forward and **fighting!** Tell me again where you are? I remember you had to travel to London, but can? it recall where from - no short term memory LOL. At least you can eat. To read of all you wonderful women **fighting** and under-going today’s treatments makes me want to reach out and hug you all and let you know that it is only the special people, with a strange I’m healthy. The unfortunate thing about all this is that you find out nobody’s too young. We’re just lucky enough to have stronger bodies to help us **fight** it. So put the boxing gloves on! And to the new Sue, I had to laugh at your family history.

I am so sorry that you’re going through this, but you need to know you’ll be fine. Yeah, it sucks, but you are doing everything you can to **fight**. You’ll be amazed at how strong you and your kids are. I’m 40 and married with two sons, 7 and 10. I can’t get over the strength. Decide and how things go for you. Welcome home. Sorry to hear about the nodes, but chemo’s not as bad as you’re probably expecting. You’ve started your **battle**, so keep **fighting**. Be good to yourself and heal quickly. You’re in my thoughts. Check my response under your other post. I was in the same position you are. Ask them!

### Results

The major hypotheses that viewing their cancer as a fight or battle would result in lowered depression post group was disconfirmed in all samples (See Table 2). Recall, significant improvement in depression was observed in all the studies used in the present examination.

In the analysis comparing the three samples, we found an ANOVA to be highly significant showing differences in a frequency of fight words. The newsgroup had twice as many fight words compared to all the other which were professionally run. Table 1 shows the means and totals for fight by each sample. An ANOVA was highly significant between sample (F=15.3, P=.001) contrast revealed high statistical significance for each sample compared to the peer sample.

## Discussion

Our study does not change the direction of the preponderance of previous studies. We believe that previous studies as well as the current one strongly support the null hypothesis. Our findings based on a here to fore unused measure of fighting spirit does add to previous studies results. We believe that assessing actual behavior rather than questionnaires is a more accurate measure of the attitude.

Our findings are in concert with prior studies that failed to find a linkage between positive outcome and an attitude of fighting spirit. Unfortunately, this attitude embedded in the culture including important organizations offer little hope for a substantial withering of this view. Despite the weight of research suggesting that fighting spirit is not necessarily a useful strategy for addressing cancer, the drumbeats of women’s magazines, fund-raising marches and some private funding agencies still promote this attitude. An interesting publication by Taut [1] examined the ethics of promoting fighting spirit as a critical factor in survival. Despite cited evidence, media and promoters of alternative medicine repeatedly stressed the importance of displaying a fighting attitude that would presumably increase the chances of a favorable prognosis. For cancer sufferers, not having fighting spirit would mean a psychological incapacity of choosing the right over the “wrong” thinking at the expense of precious months or years of survival. Put another way, it would send the message that “brave and good people defeat cancer and that cowardly and undeserving people allow it to kill them” this view can create guilt, self-flagellation and stress in woman already facing a difficult disease.

## Figures and Tables

**Table 1.** Comparison of Studies on Frequency of Fight Words

Group	Variable	N	Mean	SD	Totals
Peer Bulletin Boards	Hits	26^1^	5.31	5.47	138
	Word count	26	17,944	13,090	466,531
Life Beyond Cancer	Hits	63^2^	3.44	3.01	217
	Word count	63	6,655	1,894	419,288
Bosom Buddies	Hits	37^3^	3.27	4.62	98
	Word count	37	873.2	373.6	29,196

1. Number of members (individual scores)

2. Number of members from four chat groups conducted by the same leader (individual scores)

3. Number meeting in face-to-face chat group

**Table 2.** Regression of follow-up depression scores on fight words controlling for baseline depression and overall word count

STUDY	R^2^ CHANGE	F CHANGE	SIGNIFICANCE
PEER BULLETIN BOARDS			
Time 1 depression	0.21	8.39	.01
Word count	0.29	4.26	.05
Number fight words	0.27	0.43	.84
			
BOSOM BUDDIES			
Time 1 depression	0.27	6.37	.03
Word count	0.28	1.05	.33
Number fight words	0.24	0.41	.54
			
LIFE BEYOND CANCER			
Time 1 depression	0.27	6.37	.03
Word count	0.28	1.05	.33
Number fight words	0.24	0.41	.54

## References

[1] Taut D Is it ethical to advise people to “fight” cancer? The European Health Psychologist 2014; 16(3): 107–111.

[2] Greer S, Morris T, Pettingale KW. Psychological response to breast cancer: effect on outcome. Lancet 1979; 2(8146): 785–787. 90871 10.1016/s0140-6736(79)92127-5

[3] Watson M, Haviland JS, Greer S, Davidson J, Bliss JM. Influence of psychological response on Survival in breast cancer: A population-based cohort study. Lancet 1999; 354(9187): 1331–1336. 10533861 10.1016/s0140-6736(98)11392-2

[4] Petticrew M, Bell R, Hunter D. Influence of psychological coping on survival and recurrence in people with cancer: Systematic review. Brit Med J. 2002; 325:1066 doi: 10.1136/bmj.325.7372.1066 12424165 PMC131179

[5] Watson M, Homewood J, Haviland J, Bliss JM. Influence of psychological response on breast cancer survival: 10-year follow-up of a population- based cohort. Eur J Cancer. 2005; 41(12): 1710–1714. doi: 10.1016/j.ejca.2005.01.012 16098457

[6] Lepore SJ, Buzaglo JS, Lieberman MA, Golant M, Greener JR, Davey A. Comparing Standard Versus Prosocial Internet Support Groups for Patients With Breast Cancer: A Randomized Controlled Trial of the Helper Therapy Principle. J. Clin. Oncology 2014; 32(36): 4081–4086. doi: 10.1200/JC0.2014.57.0093 PMC4265118 25403218

[7] Winzelberg AJ, Evaluation of an Internet support group for women with primary breast cancer. Cancer. 2003; 97(5): 1164–1173. doi: 10.1002/cncr.11174 12599221

[8] Lieberman MA, Goldstein B. Self-help online: An outcome evaluation of breast cancer bulletin boards. J of Health Psychol. 2005; 10(6): 855–862. doi: 10.1177/1359105305057319 16176962

[9] Radloff LS. The CES-D Scale: a self-report depression scale for research in the general population. Appl Psychol Measure. 1977; 1(3) 385–401. doi: 10.1177/014662167700100306

[10] Zigmond AS, Snaith RP. The hospital anxiety and depression scale (HADS). Acta Psychiatr Scand. 1983; 67(6): 361–370. 6880820 10.1111/j.1600-0447.1983.tb09716.x

